# Tropical Forest Fragmentation Limits Movements, but Not Occurrence of a Generalist Pollinator Species

**DOI:** 10.1371/journal.pone.0167513

**Published:** 2016-12-12

**Authors:** Noelia L. Volpe, W. Douglas Robinson, Sarah J. K. Frey, Adam S. Hadley, Matthew G. Betts

**Affiliations:** 1 Department of Fisheries and Wildlife, Oregon State University, 104 Nash Hall, Corvallis, OR, United States of America; 2 Department of Forest Ecosystems and Society, Oregon State University, Corvallis, OR, United States of America; University of Maine, UNITED STATES

## Abstract

Habitat loss and fragmentation influence species distributions and therefore ecological processes that depend upon them. Pollination may be particularly susceptible to fragmentation, as it depends on frequent pollinator movement. Unfortunately, most pollinators are too small to track efficiently which has precluded testing the hypothesis that habitat fragmentation reduces or eliminates pollen flow by disrupting pollinator movement. We used radio-telemetry to examine space use of the green hermit hummingbird (*Phaethornis guy*), an important ‘hub’ pollinator of understory flowering plants across substantial portions of the neotropics and the primary pollinator of a keystone plant which shows reduced pollination success in fragmented landscapes. We found that green hermits strongly avoided crossing large stretches of non-forested matrix and preferred to move along stream corridors. Forest gaps as small as 50 m diminished the odds of movement by 50%. Green hermits occurred almost exclusively inside the forest, with the odds of occurrence being 8 times higher at points with >95% canopy cover compared with points having <5% canopy cover. Nevertheless, surprisingly. the species occurred in fragmented landscapes with low amounts of forest (~30% within a 2 km radius). Our results indicate that although green hermits are present even in landscapes with low amounts of tropical forest, movement within these landscapes ends up strongly constrained by forest gaps. Restricted movement of pollinators may be an underappreciated mechanism for widespread declines in pollination and plant fitness in fragmented landscapes, even when in the presence of appropriate pollinators.

## Introduction

Most plant species depend on movements of animal pollinators for directed pollen flow [1]. Habitat fragmentation alters the configuration of landscape elements and is known to affect animal movement [2–7] so pollination may be particularly susceptible to fragmentation effects [8, 9]. Dividing once-continuous habitats into patches potentially restricts how far individuals travel, because the non-habitat surrounding the native patches (i.e., the matrix) may serve as a movement barrier or reduce rates of movement between them [7]. The influence of the matrix should therefore have a strong effect on pollen flow, because the routes through which pollen moves become restricted (see [10] for examples). In such cases, the likelihood of transporting pollen among nearby genetically related plants is expected to be higher, reducing plant fecundity or resulting in inbreeding depression [11].

Although habitat loss appears to have overwhelming negative effects on species distributions, most studies to date have shown mixed effects of fragmentation per se (i.e. the arrangement of remaining patches [12]). Nevertheless, individuals may respond behaviorally to the size, shape, connectedness, and arrangement of habitat remnants [13, 14]. Therefore, it is at the level of individual organisms where the immediate effects of landscape configuration should be most evident. Fine-scale movement decisions may accumulate to generate emergent properties at broader scales, including the roles that individuals and species play as pollen and seed dispersers [15–17]. An improved understanding of how habitat configuration affects pollinator movement patterns is thus important for generating and testing hypotheses about the effects of fragmentation on pollen flow. The pollinator movement hypothesis [8] predicts that altered pollinator behavior in fragmented landscapes can lead to pollination declines even when pollinators are present [18]. Unfortunately, most pollinators are too small to track efficiently which has precluded testing this hypothesis.

In this study, we applied a large-scale mensurative experiment to determine how tropical forest loss and fragmentation influence pollinator habitat selection behavior at three spatial scales: individual point-locations, movement paths, and home ranges. We selected a common hummingbird pollinator–the green hermit (*Phaethornis guy*)–due to its role as a pollinator of a large number of understory tropical plant species [19, 20], particularly *Heliconia tortuosa*, a keystone herb we have previously shown to be fragmentation sensitive as exemplified by its depressed rates of reproduction in small fragments [21]. Low rates of seed set despite high pollen deposition suggest that fragmentation may be reducing the quality of pollen delivered [21]. Under the pollinator movement hypothesis, these low rates of seed set could be due to green hermits showing behavioral aversion to crossing gaps and select movement paths that minimize exposure to open areas during daily within-home range foraging [22–24].

## Materials and Methods

We conducted this study in a 20,600 ha area surrounding the Las Cruces Biological Station, Costa Rica (8° 47’ N, 82° 57’ W; *ca*. 900–1400 m elev.). The landscape was previously forested, but now remnant fragments of Pacific premontane humid forest (1–1400 ha) and forested riparian corridors (10–40 m wide) are scattered through agricultural matrix, comprised of pasture (>90%), mixed coffee-banana plantations (~5%) and family garden plots [5, 20]. We delineated land cover for the entire study region into forest, pasture, or non-pasture matrix (i.e., banana, coffee) [25] using ArcGIS 10.1 [26].

We captured 20 individuals (8 male, 12 female) at forest fragments chosen according to a stratified random sampling design to represent a gradient in patch size (1.47–800 ha) and forest amount (16–78%) within a 1-km buffer from the focal patch (sensu [27]). By randomly sampling within four categories (small patches in landscapes with low amounts of forest, small patches in landscapes with high amounts of forest, large patches in landscapes with low amounts of forest and large patches with high amounts of forest) we were able to insure the full variation was represented. This mensurative experimental approach enabled us to disentangle the effects of landscape composition (i.e., forest amount, pasture) and landscape fragmentation (i.e., patch size, connectivity). Green hermits are thought to be strongly forest associated [5]. The 1-km distance corresponds to the maximum observed daily movement distance based on pilot data [25]. Green hermits do not defend territories but instead are thought to exhibit ‘traplining’ behavior, moving relatively long distances to feed from nectar-rich flowers [28]. Birds were captured with mist-nets set near food sources (mainly *Heliconia* sp. and *Centropogon* sp.), and with hall traps containing a hummingbird feeder. Captures were done between 6:00 and 12:00hs. Each individual was banded and fitted with radio-telemetry units (<0.25 g, Blackburn Transmitters), using eyelash glue for attachment to bare skin on their lower backs (sensu [5, 25]). During the process, the individuals were regularly fed sugar-water to ensure recovery from the capture process. Green hermits are relatively large hummingbirds (5.8 ± 0.09g), so transmitter mass was <5% of their body mass. The attachment of transmitters did not appear to affect the behavior of tagged individuals as radio-tagged birds were observed carrying on normally with their reproductive behavior (chasing competitors, lek display, nesting and feeding). Research protocols, including capture, banding and transmitter attachment, have been approved by Oregon State University Institutional Animal Care and Use Committee (IACUC–protocol number 4265). All sites were accessed with permission from Ministerio de Ambiente y Energía, (MINAE).

We radio-tracked hermits from March to May, 2012. This represents the dry season for this region. Each day the initial radio fix was obtained by searching for the transmitter signal from the closest road near the last observed location of the bird. These locations were in open areas, which allowed us to make a first assessment of whether the birds were inside or outside forest patches. We then obtained locations by following birds as closely as possible on foot using radio receivers and handheld Yagi antennae. We gathered at least 6 hours of observations per bird (mean = 14.4 h, SD = 3.9) in continuous bouts over a period ranging from 2 to 8 days (mean = 4 days / individual). The combined number of sampling hours amounted to 288 h, during which 2428 individual locations were recorded. For analyses, we used only data acquired from a distance of less than 30-m from the bird (46% from 0-10m; 32% from 11-20m; 22% from 21-30m), as these were the records with a higher degree of certainty (*N* = 1561). At each point where the bird was located, observers registered UTM coordinates using a GPS device (Mean accuracy: 6.54 m) [25].

### Point-scale data analysis

#### Forest dependency

We compared forest amount at observed locations to the amount present in the area that we expected to be available to each individual. To characterize ‘used’ sites, we generated a 30-m buffer around each recorded point [29]. ‘Available’ was defined as the proportion of forest within a 500-m buffer around each recorded point (500 m is considered available based on the observation hermits can fly at least 500 m without stopping; Volpe unpublished data). We used ArcGIS 10.1 to create the buffers and calculate the percentage of forest inside them. To prevent giving excessive weights to location points with multiple records, we only used records separated by >1 m. The final dataset contained 1359 points. We expected that the amount of forest within used buffers to be larger than the amount available in the surrounding area. To test this prediction we applied the linear mixed effects model:

Difference=TotalForest|Individual

where ‘Difference’ corresponds to the difference between observed and available percentage of forest within the buffers. The use of this response allowed us to pair observed and available locations. ‘Total forest’ was defined for each bird as the percentage of forest available within the surrounding 500 m. This variable was included to prevent a bias towards observing larger differences between used and available forest for birds living in areas with low overall forest amount. We included ‘individual’ as a random intercept effect to account for potential lack of independence within points selected by each bird. We used the *ncf* R package to generate a correlogram of the residuals, which showed the existence of spatial autocorrelation (Moran’s I values >0.1). To account for this, we generated models with four different spatial autocorrelation structures: spherical, gaussian, rational quadratic and exponential. A comparison of the models’ AICc showed that the best one included a rational quadratic autocorrelation structure [30]. The test was applied using the ‘*nlme’* R package [31].

#### Resource availability and canopy cover

To acquire data suitable for examining importance of vegetation structure and resource availability, we measured vegetation characteristics within 20 m radius plots surrounding detection locations (number of locations per bird: range = 8–18; mean = 13.4). Time limitations prevented us from collecting data on resources available to all tagged individuals so we worked on a subset of birds (n = 15) that still represents a gradient in forest loss and fragmentation. Each of the observed locations for these 15 birds was paired with a random sample plot located within 500 m of the original observation. In each plot we recorded canopy cover and food resource availability. Canopy cover refers to the area of the ground covered by a vertical projection of the canopy, reflecting the dominance of a site by trees [32], and was measured using an ocular tube following the approach of Kucharcik and Collins [33]. Resource availability was measured by counting the number of flowering plants within the limits of the plots [21]. There was no evidence of correlation between the two variables (Pearson’s correlation = 0.219). Including both factors in the model helps to disentangle their effects as it allows us to predict how resources affect the likelihood of presence given a certain amount of forest cover and vice-versa.

To test the hypothesis that green hermits prefer locations with high canopy cover and resource availability, we applied a logistic mixed-effect regression model that compared ‘used’ versus ‘available’ green hermit locations:

Presence=Resourceavailability+Canopycover|Pair/Individual

‘Presence’ indicates if a particular point was used or not by a bird at a given time. Resource availability refers to the number of flowering plants within the 20-m radius plot. Only plants belonging to the family Heliconiaceae were used for the analysis as these are the most common and nectar-rich ornithophilous plants in our system. Results were qualitatively similar when we included all flowering ornithophilous plants in the analysis. We included ‘pair’ nested within ‘individual’ as random intercept effects in order to both pair the observed-random plots and to account for potential lack of independence within points selected by each bird. The tests were applied using the ‘*lme4’* R package [31].

### Path-scale data analysis

We used a step selection function (hereafter ‘SSF’ [34]) to assess the hypothesis that green hermits actively select forest during movement. This technique compares the straight line (‘step’) connecting consecutively visited points with alternative steps that were not taken but originate at the same point (hereafter ‘available’ steps). The model assumes that environmental characteristics along the lines are correlated with the probability of moving to a particular end point [34]. For each bird, we generated alternative random steps based on the frequency distribution of the step lengths and turning angles observed for the *remaining* birds, using the function ‘movement.ssfsamples’ from the program GME (Geospatial Modelling Environment [35]). We generated 20 random steps per observed step, following Gillies, Beyer, & St Clair [16]. We resampled the data to obtain origin-destination pairs separated by a distance long enough to be able to provide information about the area around them (10 meters) but close enough in time to not be completely unrelated (15 minutes). These constraints resulted in 903 ‘used’ steps. To ensure that the available steps were realistic, we only used those that ended in forest habitat.

We used a mixed matched case-control logistic regression (also termed ‘mixed conditional logistic regression’ [36]) to model the likelihood of an individual hummingbird choosing a particular movement step instead of an alternative. To find the best model, we first identified four ‘exposure variables’, defined as variables that influence the level of exposure of the individuals to unfavorable conditions [16]. Variables we expected to increase exposure were: number of gaps per step, total gap distance and mean gap size. Variables expected to reduce exposure were: total forest and proportion of forest within a step. To test for the effect of matrix type (e.g., pasture, non-pasture) on movement decisions we included an interaction between exposure variables and the proportion of the linear dimension of a gap that occurred in pasture. Due to the structural complexity of vegetation in non-pasture matrix (i.e., coffee, banana plantation) we expected this cover type to be more permeable than pasture. We also included a variable we expected to facilitate movement–distance to the nearest stream. Our previous work indicates that green hermits may use streams as movement corridors [25].

To select the most parsimonious models for path-scale selection, we built competing candidate models and compared these using Akaike’s Information Criterion corrected for small sample size (AIC_C_). We applied the mixed conditional logistic regressions in the *mixlogit* module [37] in Stata [38] to obtain both the models and the AICc values. Each set of candidate models included a univariate model with a single exposure variable and a full model including the exposure variable and distance to stream. In models that included number of gaps, mean gap size or total gap distance, we also introduced an interaction term between these and the pasture covariate. We expected that gaps consisting of pasture would have a stronger effect than a non-pasture matrix, as the birds can potentially find cover and food resources in the latter (e.g.: crops).

All models included a random component that allowed variation among individuals in the selection coefficient for each variable [36]. To test if there were specific factors affecting the selection decisions of individuals, we used linear regression to predict individual selection coefficients as a function of broad-scale landscape measures (i.e., overall forest availability, connectivity within the home range) and individual-specific characteristics (i.e., sex).

We measured connectivity using the Landscape Coincidence Probability Index [39]:

$$CON=\sum_{i=1}^{NC}\sum_{j=1}^{NC}(\frac{a_{i}a_{j}c_{ij}}{A_{L}^{2}})\times 100$$

where NC is the total number of forest patches, a_*i*_ and a_*j*_ are areas of patches *i* and *j* respectively, c_*ij*_ is a ‘passibility’ value which is 0 or 1, and A_L_ is the total landscape area within the home range (including both forest and non-forest patches). Patches were considered fully connected (c_*ij*_ = 1) if they were within the maximum observed gap-crossing distance (162 m; n = 9, mean = 84.5 m) and unconnected (c_*ij*_ = 0) if they were separated by more than that distance. We chose this metric because it quantifies not only the capacity of organisms to cross between forest patches but also quantifies patch area in relation to the total landscape area. In this way, maximum levels of connectivity were assigned to those landscapes in which all the forest was contained in the same patch and this patch occupied a large proportion of the total area. We expected birds living in areas with high forest amount and connectivity to show a greater tendency to avoid open areas, because they have more forested alternatives available.

### Home range-scale data analysis

We generated home range estimates using the Local Convex Hull (hereafter ‘LoCoH’) approach, with home range defined as the area encompassed by 95% of the points. We chose LoCoH because it is sensitive to sharp boundaries in habitat use [40], such as the ones we observed in this predominantly forest-pasture system. LoCoH home range polygons where generated with the ‘*tLoCoH*’ R package, using the *k*-method. To determine the sensitivity of the results to home range estimation methods, we also generated polygons using the Brownian Bridge Movement Model (hereafter ‘BBMM’ [41]). Though mean home range size was twice as large using this method, overall results did not differ qualitatively so we report only LoCoH results.

To evaluate the hypothesis that green hermits select home ranges with more forest and greater connectivity, we rotated LoCoH home range polygons around their center (degree of rotation = 1; n = 359/bird) to simulate potential areas that were available to hummingbirds, and calculated (1) percentage of forest (2) connectivity, and (3) mean patch size, inside both observed and hypothetical scenarios (hereafter ‘rotated alternatives’). As the data were not normally distributed, we applied a paired Wilcoxon signed-rank test to compare the observed percentage of forest versus the mean value for rotated alternatives. We excluded cases where both observed and all rotated alternatives fell completely inside forest, reducing the sample size to 17 individuals.

Finally, we tested whether amount of forest and a metric of fragmentation (mean patch size; MPS) influenced home range size. Both variables were measured within circular buffers with an area equal to the one of the largest home range polygon. We selected mean patch size as a measure of fragmentation, which is a commonly used surrogate given its straightforward interpretation in forest versus non-forest situations [42]. For example, in two landscapes with similar forest amounts, smaller mean patch size indicates higher levels of subdivision. We then modeled home range area as a function of forest amount, mean patch size (MPS) and sex:

log(HRarea)=Forest%+MPS+Sex

‘Forest %’ refers to the proportion of the circular buffer covered by forest and MPS refers to the mean patch size within the home range. Our expectation was that increasing fragmentation limits the degree to which home ranges can expand, even when the total amount of forest available in the landscape is high. We log-transformed home range area to meet the regression assumptions. MPS and forest amount were not highly correlated, so we included them in the same model (Pearson’s correlation = 0.12)

## Results

### Point-scale habitat selection

#### Forest dependency

We found that green hermits selected locations at the point scale with an average of 42% more forest than in available surrounding landscapes (CI 95%: 32.3% to 52.1%, *P* <0.0001). The relationship was statistically significant even after controlling for the negative relationship between total amount of forest and difference between observed and available use of forest, although 95% CI narrowly overlapped zero (-0.28%, CI 95%: -0.53 to 0.043%, *P* = 0.02).

#### Resource density and canopy cover

**Fig 1:**
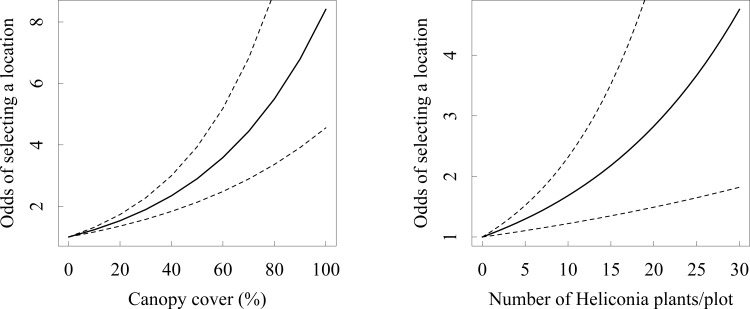
Effect of canopy cover (A) and number of Heliconia plants (B) on the odds of the green hermit hummingbird choosing a given location, as calculated from the point-level regression model.

The presence of green hermits at a given point was positively related to both canopy cover and resource availability. The addition of one flowering plant to a 0.13 ha plot increased the chances of green hermit visits by a factor of 1.054 (95% CI: 1.020 to 1.090). This translates to approximately doubling of the chances of site-level selection for each increase by 15 plants (or 119 plants/ ha, Fig 1A). Similarly, an increase of 1% in canopy cover increased the likelihood of site use by a factor of 1.022 (95% CI: 1.015 to 1.028). A plot with 100% of canopy cover was 8 times more likely to be used than one with no canopy (Fig 1B). A standardized comparison showed that canopy cover was the most important variable determining point-level habitat selection (β_canopy_: 0.84, β_Resources_: 0.56).

#### Path-scale habitat selection

**Fig 2:**
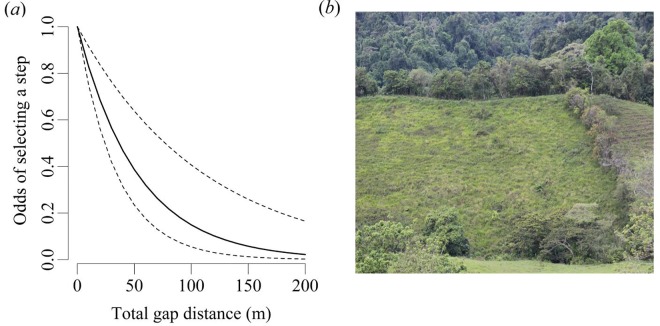
(a) Effect of total gap distance on the odds of the green hermit hummingbird choosing a given step, as calculated from the top step selection function model provided in Table 2. (b) Large gaps in tropical forest are unlikely to be crossed.

Green hermits selected movement steps that decreased their exposure to gaps in forest cover. All candidate models showed that variables decreasing exposure (i.e., forest amount, proportion of step in forest) had clear positive effects on path choice, while variables that increased exposure (i.e., number of gaps, mean gap size and total gap length) had negative effects (Table 1). Green hermits selected steps that took them closer to streams and avoided those crossing long stretches of open matrix (Fig 2, Stream distance: SD = -0.013, Z = -5.66, *P*<0.001, total gap length: SD = 0.03, Z = 6.22, P<0.001). None of the interactions between pasture and exposure variables were statistically significant.

**Table 1:** Model coefficients, standard errors, confidence intervals, odds ratios and AICc values for the candidate models used to predict observed hummingbird movement steps in relation to random unused steps as a function of the following variables. The top-ranked AICc model is bold.

Model	Variable	Coeff.	SE	Ci	Cs	OR	AICc	ΔAICc
**Stream + TotGap**	Stream	-0.020	0.003	-0.026	-0.014	0.981	5218	0
	TotGap	-0.019	0.005	-0.029	-0.009	0.981	5218	
Stream + TotGap * Pasture	Stream	-0.0181	0.003	-0.024	-0.012	0.982	5224	6
	TotGap	-0.010	0.004	-0.018	-0.002	0.990	5224	
	TotGap *Pasture	-0.005	0.006	-0.017	0.007	0.995	5224	
Stream + MeanGap	Stream	-0.019	0.003	-0.026	-0.013	0.981	5229	10
	MeanGap	-0.020	0.006	-0.032	-0.008	0.980	5229	
Stream + MeanGap * Pasture	Stream	-0.017	0.003	-0.023	-0.011	0.983	5242	24
	MeanGap	0.003	0.004	-0.005	0.011	1.003	5242	
	MeanGap *Pasture	-0.007	0.007	-0.020	0.007	0.993	5242	
TotGap	TotGap	-0.033	0.009	-0.051	-0.015	0.968	5320	101
Stream + PropInFor	Stream	-0.017	0.003	-0.023	-0.011	0.983	5322	104
	PropInFor	0.779	0.501	-0.223	1.781	2.180	5322	
Stream + NumGap * Pasture	Stream	-0.0177	0.003	-0.024	-0.012	0.982	5332	114
	NumGap	-0.218	0.225	-0.668	0.232	0.804	5332	
	NumGap *Pasture	-0.726	0.620	-1.966	0.514	0.484	5332	
Stream + ForAm	Stream	-0.021	0.004	-0.029	-0.013	0.979	5333	114
	ForAm	0.353	0.253	-0.153	0.859	1.423	5333	
Stream + NumGap	Stream	-0.018	0.005	-0.028	-0.009	0.982	5334	116
	NumGap	-0.169	0.281	-0.731	0.393	0.845	5334	
MeanGap	MeanGap	-0.042	0.011	-0.064	-0.02	0.959	5338	120
Stream	Stream	-0.018	0.004	-0.026	-0.009	0.983	5358	139
PropInFor	PropInFor	1.702	0.849	0.004	3.399	5.484	5415	197
NumGap	NumGap	-0.539	0.324	-1.189	0.109	0.583	5433	215
ForAm	ForAm	0.523	0.320	-0.117	1.163	1.687	5449	231

**SE**: standard errors, **Ci**: confidence intervals, **Cs**: confidence intervals, **OR**: odds ratios, **Stream**: distance to stream, **TotGap**: total gap length along a step, **ForAm**: percentage of forest inside a buffer surrounding the step, **PropInFor**: proportion of the step in forest habitat, **NumGap**: number of gaps along the step, **MeanGap**: mean gap size, **Pasture**: proportion of the gap that takes place inside pasture,

* Indicates an interaction between two variables.

The tendency to select paths near streams did not appear to be mediated by features that were specific to individual hummingbirds (i.e., sex, total forest available or connectivity) (Table 2). We did, however, find a weak negative effect of connectivity on use of large gaps; an increase in the connectivity index decreased use of open areas (β = -0.00052; CI 95%: -0.0003 to -0.0007).

**Table 2:** Final models predicting the individual-specific coefficients for total gap length and distance to stream from the step selection function (SSF) models. None of the explanatory variables (forest amount, connectivity and sex of the bird) were able to explain the variability observed in the selection coefficients for stream distance, while only connectivity affected the selection coefficients for total gap length.

SSF variable	Variable	Coefficient	SE	P
**Total gap length**	Intercept	0.01	0.008	0.0925
	Connectivity	-0.00052	0.00010	0.0001
**Stream distance**	Intercept	-0.017	0.003	<0.0001

### Home range scale habitat selection

The size of home ranges varied from 0.25 ha to 6.08 ha (mean = 2.18 ha), with home ranges of males tending to be larger (mean = 3.12 ha) than those of females (mean = 1.50 ha). Mean home range length was 282 m (SD = 179 m, median = 240 m, range = 41 m– 687 m). These values exclude an outlier whose estimated home range area and length were 153 ha and 2883 m respectively; no other individual was detected farther than 770 m from its capture site.

Across all green hermits, an average of seventy-seven percent of home range area was forested (median = 82%, range = 32% - 100%). Percentage of forest inside home range polygons tended to be larger than the amount available in the surrounding landscape (Wilcoxon signed rank test; V = 146, *P* = 0.0003). The mean difference in forest between observed and rotated alternatives was 10.7% (SE = 0.61). When excluding cases where the availability only included forest (n = 4), the difference increased to 13.3%.

Connectivity within the home range polygons was significantly higher than connectivity within rotated alternatives (*P* = 0.001, t = 3.9, df = 19). The connectivity index within observed home ranges was, on average, 13.4% greater than available (CI 95% = 6.1% to 20.6%). However, when we repeated the comparison using only available home ranges that had similar amounts of forest as in observed home ranges (+/- 5%), the difference was not statistically significant (*P* = 0.78, t = 0.28, df = 15). In this case, connectivity within observed home ranges was only an average of 0.12% larger than those available (CI 95% = -0.8% to 1.02%). Using this more conservative approach, the positive effect of connectivity was indistinguishable from the effect of forest amount.

#### Home range size

We conducted analyses at two scales, using two buffer sizes corresponding to the maximum home range area including and excluding the outlier: 153 ha and 6.08 ha respectively. We found no evidence for an effect of mean patch size (buffer 153 ha: β = 0.02, P = 0.53, buffer 6.08 ha: β = -0.008, P = 0.94), forest amount (buffer 153 ha: β = 1.99, P = 0.14, buffer 6.08 ha: β = -0.036, P = 0.67) or sex on home range size (buffer 153 ha: β = 1.24, P = 0.05, buffer 6.08 ha: β = 0.77, P = 0.109). The values presented here correspond to univariate models.

## Discussion

At the scale of the home range, habitat selection by green hermits appears to be dependent largely on the amount of tropical forest rather than its configuration. Degree of landscape connectivity, independent of forest amount, appeared to have little effect on home range selection. These results are consistent with earlier work showing that habitat amount is often thought to exert a stronger driver of species occurrence than habitat configuration [12, 43, 44]. Similarly, at the point level green hermits occurred in sites with a high amount of forest canopy cover.

However, at the path level, green hermits chose to avoid gaps in forest caused by agriculture and, when possible, moved primarily within closed canopy forest. Indeed, gaps as small as 50 m reduced the odds of movement by half. The pollinator movement hypothesis posits that despite lack of fragmentation effects on pollinator abundances, fragmentation limits plant reproduction via reduced (or altered) pollinator movement patterns [8]. Our results at the movement path scale support this hypothesis. Because hermits moved across gaps between remnant forest patch at reduced rates, long-distance pollen flow should be infrequent, which may decrease the likelihood of high-quality outcrossed pollen reaching plants in isolated fragments. Indeed, our previous results indicate that for *H*. *tortuosa* (a species for which green hermits are a primary pollinator; [19]), isolated patches [21] despite adequate pollen delivery suggesting that lower quality pollen is being delivered. Strong avoidance of open areas at the path scale should result in greater rates of pollen flow in connected versus unconnected patches [45] with implications for heterozygosity in the short term, and ultimately the capacity for plants to respond to environmental change [11]. Conservation policies that promote forest connectivity are likely to be vital for the maintenance of healthy ecosystems by promoting increased gene exchange among flowering plants [13]. The strong effects of forest configuration on hummingbird movements seems at odds with the fact that we found no effect of forest fragmentation per se on hummingbird home range location or home range size. One would expect such movement effects to scale-up to influence home range characteristics; we predicted that home ranges in severely fragmented landscapes would be smaller due to movement limitation (with subsequent impacts on pollen flow). In our system, hummingbirds seem to select landscapes with sufficient forest cover (~30%) such that patches within home ranges are still relatively well connected. We predict that this apparent behavioral capacity to buffer fragmentation effects via home range selection is likely to decrease if the amount of tropical forest continues to decline. Indeed, the theoretical and empirical literature predicts such “fragmentation thresholds” at low (<30%) amounts of native habitat [46, 47].

The tendency for hummingbirds to select movement paths associated with forest adds to the growing body of evidence showing the importance of forested landscape elements to animal movements. For instance, riparian corridors are thought to increase connectivity between patches by acting as thoroughfares between resource patches [48]. In our study, green hermits showed a marked preference for following streams. Stream corridors are some of the last remaining areas of forest cover in agricultural landscapes of the Neotropics. We also observed a preference for streams in unfragmented forest, suggesting that additional factors associated with riparian zones guide hummingbird behavior. Streams may facilitate movement by acting as ‘flight-paths’ [49], offering open pathways through otherwise dense forest. Alternatively, there may be a higher abundance of floral resources or nesting sites in damp areas next to streams [28]. Regardless of the mechanism, the presence of riparian corridors in fragmented areas appears to facilitate movements among otherwise unconnected sites.

Surprisingly, we found no evidence for an effect of non-forest matrix type on movement decisions by the green hermit. Pasture appeared to restrict movement no more than other forms of non-forest matrix (e.g., coffee and banana plantation), despite offering no resources or any variation in vegetation structure. However, gaps in forest are more likely to act as a filter than an absolute barrier. We observed gap crossing by green hermits traveling between two unconnected forest patches that were 129 to 162 m apart. This physical capacity to cross non-forested areas explains how green hermits are sometimes present in small patches completely surrounded by non-forest matrix [21]–particularly if one-time natal dispersal movements are less sensitive to gaps than chronic foraging movements of adults [50–52].

The structure of plant-pollinator networks is expected to buffer pollination against changes in landscape structure [8, 53], as the disappearance of a particular pollinator can be compensated by other species that maintain network connections [54]. Mobile generalist pollinator species are thought to be particularly important in this buffering process. The fact that the green hermit, a generalist pollinator ‘hub’ species [55] is affected by landscape structure despite its high vagility and capacity to persist in disturbed areas, suggests the role of forest fragmentation in the destabilization of pollination networks may be greater than previously anticipated. In our system, this risk of pollination web collapse is particularly acute given the strong dependence by *H*. *tortuosa* (a keystone hub plant species) on visitation by only two species of traplining hummingbirds for pollination [19].

In conclusion, we found that space-use by green hermit hummingbirds was strongly associated with forest amount at all scales examined. Despite this, green hermits persisted in landscapes with surprisingly low forest cover. Nevertheless, persistence in fragmented areas does not imply indifference; movement patterns of green hermits were still affected by characteristics of landscape elements. These results suggest that plant gene flow in fragmented landscapes could be reduced despite the presence of adequate pollinator abundance. Restricted movement by pollinators may be an underappreciated mechanism for widespread declines in pollination and plant fitness.
